# VGDS-PointNet++ for organ segmentation and phenotypic trait estimation in greenhouse tomato seedlings

**DOI:** 10.3389/fpls.2026.1753706

**Published:** 2026-04-13

**Authors:** Feiyi Wang, Tianyu Zhu, Lyuwen Huang, Ce Feng, Xi Lu, Wenchong Min, Zheng Wang, Xiaohui Hu, Yanming Nie

**Affiliations:** 1 College of Information Engineering, Northwest A&F University, Xianyang, Shaanxi, China; 2 Key Laboratory of Agricultural Internet of Things, Ministry of Agriculture and Rural Affairs, Xianyang, Shaanxi, China; 3 Shaanxi Engineering Research Center of Agricultural Information Intelligent Perception and Analysis, Northwest A&F University, Yangling, China; 4 College of Horticulture, Northwest A&F University, Xianyang, Shaanxi, China

**Keywords:** leaf length calculation, organ segmentation, space curve fitting, tomato canopy, VGDS-PointNet++

## Abstract

**Introduction:**

To accurately segment point clouds and quickly calculate leaf length and stem diameter, thereby enabling phenotypic analysis and variety selection of greenhouse tomato plants, this paper proposes a voxel grid downsampling (VGDS)–PointNet++-based model for point cloud segmentation and trait calculation.

**Methods:**

The point clouds of the tomato canopy were acquired using a depth camera. After labeling, point cloud augmentation was performed, and the tomato point cloud dataset (TPCD) containing 1,552 sets of data was rebuilt. Voxel grid downsampling was applied to replace the original sampling strategy of PointNet++. Models of PointNet, PointNet++, VGDS-PointNet++, and Point Transformer were trained with the TPCD and compared on segmentation quality with accuracy and mean Intersection over Union (mIoU). After segmentation, skeletal morphology was fitted for non-occluded leaves by applying a series of surface fitting techniques. The leaf lengths and stem diameters were automatically calculated and compared with the manually measured values.

**Results:**

The validation results showed that the average runtime of voxel grid downsampling was 0.132 s, which was lower than under the same number of sampled points. Compared to the other three models, the proposed model had higher accuracy and mIoU, reaching up to 96.80% and 88.95%, respectively. The proposal’s accuracy and mIoU increased by 3.9% and 4.45% over PointNet++, respectively. The determination coefficient *R*^2^ between the automatic calculation and manual measurement values of leaf length and stem diameter was 0.93 and 0.87, respectively.

**Discussion:**

This can help extract phenotypic traits of tomatoes using depth cameras.

## 1 Introduction

During tomato cultivation, non-destructive monitoring of organ-level phenotypic traits provides a critical approach for assessing plant growth and health status (Jang et al., 2025; Polder et al., 2024). Due to the high subjectivity of manual measuring, the use of measuring instruments can cause irreversible damage to tomato plants. The use of depth sensors to collect point cloud data (PCD) has been recognized as an effective, convenient, and non-contact way of measuring phenotypic traits (Ma et al., 2024a). However, after acquiring PCD, it remains challenging to develop a general method for quickly and accurately segmenting tomato organs and extracting phenotypic traits. At present, some deep models, such as PointNet++ (Yang et al., 2025), Point Transformer (Xie et al., 2025), and PointNet, have been proposed for the segmentation of PCD, which have provided a theoretical basis for organ detection and segmentation of greenhouse crops. As for the phenotypic trait calculation of greenhouse tomato seedlings, this remains difficult due to variable plant growth posture. Here, a new segmentation model of deep learning, optimized with PointNet++ and voxel grid downsampling, is proposed.

For the organ segmentation of greenhouse crops of PCD, different deep models have been applied for different crops (Rahman et al., 2025; Yao et al., 2025). To address this issue, Zhong et al. (2025) enhanced the characterization capability of the PointNet++ network by incorporating features related to centroid and proximity relationships. Nevertheless, the computational efficiency of the model was relatively low due to the prolonged time required to extract the neighborhood relationship and centroid features. Particularly, the segmentation of leaves in greenhouse crops, such as cabbage and lettuce (Chen and Lai, 2025), was complicated by the presence of highly overlapping and similar leaves, which presented a challenge for accurate recognition. The incorporation of Attention-Based State Space Abstraction (ASAP) attention into PointNet++ enabled it to process data with an uneven distribution, thereby improving the computational efficiency of feature-rich regions. This, in turn, facilitates the accurate recognition of plant organs (Bekkemoen and Langseth, 2024). The integration of local feature aggregation and a self-attention module in the PointNet++ network structure enhanced the model’s ability to interact with neighborhood information, improved the recognition performance, and obtained the pathological features of crop, thus more accurately predicting and evaluating maize cob lesions (Yang et al., 2024). It had been verified that the time cost of the PointNet++ sampling strategy was proportional to the number of point clouds (Wang et al., 2025). Consequently, for large-scale point clouds, the time complexity should be reduced to meet the application requirements while ensuring the recognition accuracy of crop organs. The segmentation outcomes serve as a solid foundation for calculating phenotypic parameters.

The phenotypic trait parameters of plant organs, as important reference values for assessing health states during the growth cycle, need to be effectively segmented and accurately computed. Usenko et al. (2025) reconstructed the 3D shape of the plants using a machine learning model to learn the complex mapping relationship between 3D geometric forms and the total leaf area, and they obtained the overall leaf area values of the tomato plants. Qiao et al. (2025) used Principal Component Analysis (PCA) and projection methods to calculate the plant height, leaf length, and leaf width of corn plants. By combining the least squares method and linear fitting, they obtained the stem thickness, effectively capturing and reflecting the actual characteristics of the plants. Jia et al. (2025) combined the Hilditch algorithm and the Hough transform algorithm to obtain the transformation relationship between the actual length and pixels of the detection points of the seedlings. Using coins as the scale between the actual values and the pixel values, they calculated the actual length of the rice seedling buds and provided accurate length detection results. Even the recent algorithms based on a deep learning network to segment objective plants do not meet accurate requirements after training when dealing with other complex or diverse plant structures.

**Figure 1:**
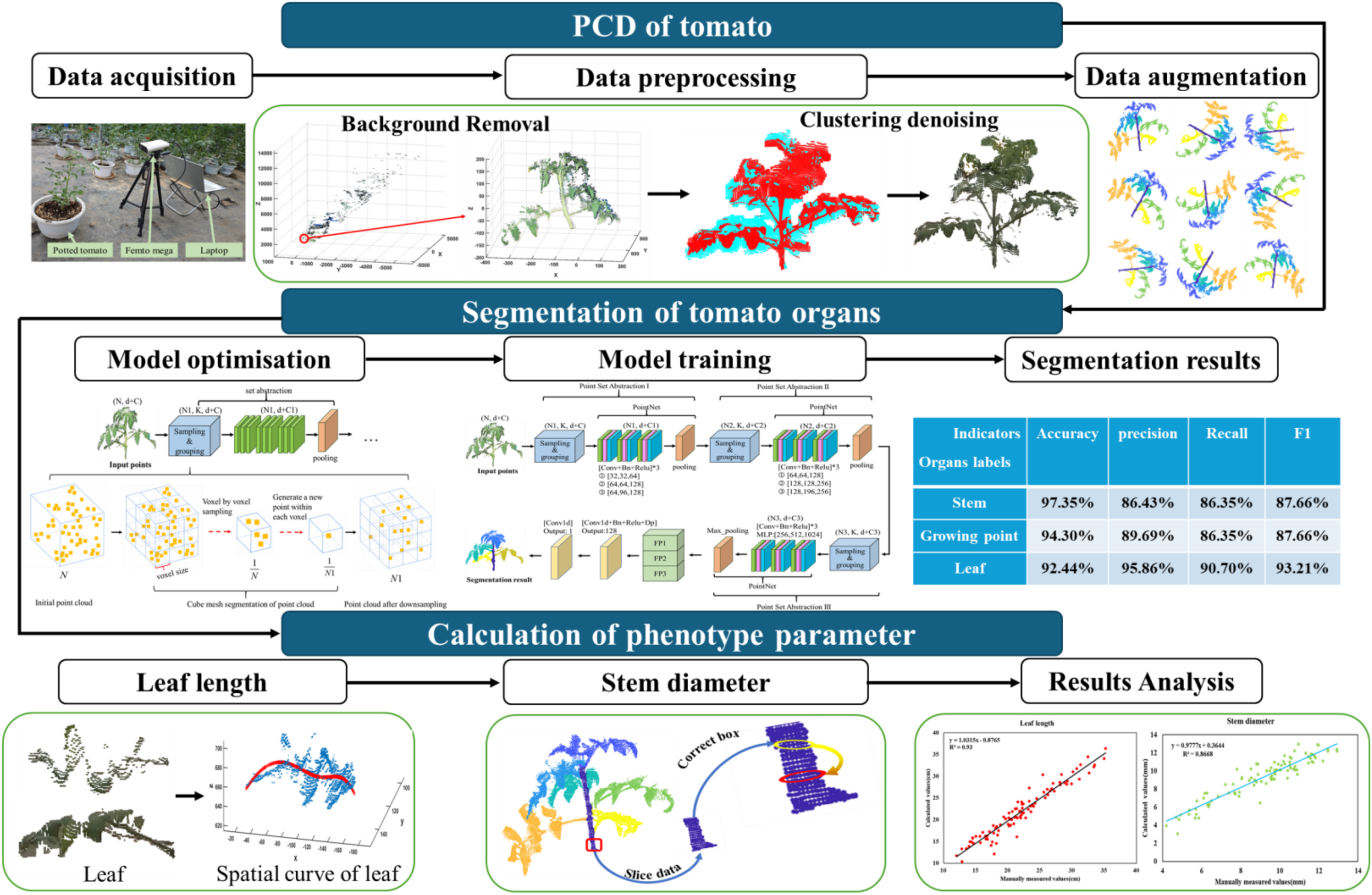
Overview of the proposed pipeline.

With regard to these issues or challenges, a framework is proposed for organ recognition and segmentation, as shown in **Figure 1**. The figure consists of three parts. The first part is the PCD of tomato, which involves the acquisition of tomato data and the establishment of the dataset. The second part is the segmentation of tomato organs, where the model structure of PointNet++ is improved, the sampling method and the learner are replaced, and the performance of the model is evaluated. The third part is the calculation of the phenotype parameter. It includes the calculation of stem thickness and leaf length, and the results are analyzed. After obtaining the point cloud of tomatoes using a depth camera, the tomato point cloud dataset (TPCD) was established through enhancement, labeling, and other preprocesses. The Set Abstraction (SA) module of PointNet++ was optimized, and a new voxel grid downsampling (VGDS)–PointNet++ (voxel grid downsampling–PointNet++) was constructed. The TPCD (tomato point cloud data) was used for the training of an improved PointNet++ model. The leaves, growth points, and stems were segmented by the proposal. The trait parameters of leaf and stem diameter were calculated by the proposed plane fitting, spatial curve fitting, and spatial projection.

The rest of the paper is organized as follows. Section 2 describes the methodology for obtaining and generating the PCD of tomato, organ segmentation with an optimized deep model, leaf length, and stem calculation. Section 3 discusses experimental results and provides a discussion. The last section summarizes the paper and extends future work. The main contributions to the proposal are as follows:

1. To precisely segment tomato organs, a new VGDS-PointNet++ segmentation method was proposed to enhance training efficiency and segmentation accuracy. Here, the sampling strategy of the SA (set abstraction) hierarchical structure was redesigned, and the learner was replaced in PointNet++. This resulted in improved segmentation efficiency and accuracy of the organs.
2. To accurately obtain the phenotypic parameters of leaf length and stem diameter, the skeleton structure of the leaf veins was extracted using the surface fitting method, and the curve segments were fitted to extract the leaf length. The direction was corrected, and the cross-section of the stem was cut to extract the stem diameter. This solved the problem of calculating the leaf length and stem diameter through cloud computing with a single viewpoint in a greenhouse environment.
3. To enhance the diversity of the point cloud data, a tomato plant point cloud dataset for training deep learning models was constructed using data augmentation methods.

## 2 Materials and methods

### 2.1 Data acquisition

As shown in **Figure 1**, the collection site was located at the horticultural field of Northwest A&F University (34°17′33″N, 108°4′27″E), which has a temperate continental monsoon climate. The tomato variety was Heart Sweet. Tomatoes are annual or perennial herbs. They have climbing stems and require support, such as a trellis, after flowering to facilitate vertical growth. Tomatoes thrive in warm climates, with an optimal growth temperature range of 23 °C–28 °C (Kürklü et al., 2025).

A Femto Mega depth camera (ORBBEC Technology Group Co., Ltd., Shenzhen, China) was used to acquire RGB-D images and generate point clouds. The sensor was based on time-of-flight (ToF) technology with a working distance of 0.3–3 m. During acquisition, the depth image resolution was set to 640 × 576 pixels and the RGB image resolution to 3,840 × 2,160 pixels. The coordinate unit of the acquired point clouds was in millimeters (mm), as defined by the depth sensor calibration. Each PLY file contained approximately 5 million points.

The study selected potted tomatoes in the seedling stage under greenhouse conditions as the data source. To reduce the interference of solar radiation on the laser camera, the experiment was conducted between 09:00 and 10:30 in the morning. This time period has more diffuse light, better imaging results, and relatively fewer noise points. Data collection was conducted from October to December 2024 under a greenhouse. To ensure adequate coverage of leaf morphology in a single-view acquisition setting, the depth camera was mounted in a downward-facing configuration. The inclination angle relative to the ground ranged from 0° to 30°, with the camera positioned 50–65 cm above ground level. The distance between the camera lens and the plant canopy was maintained between 35 and 45 cm. A total of 200 independent point cloud samples were collected. For this study, 97 samples corresponding to a uniform vegetative growth stage (approximately three to seven true leaves) were retained to ensure consistent organ morphology and comparable structural characteristics for segmentation and phenotypic analysis.

**Figure 2:**
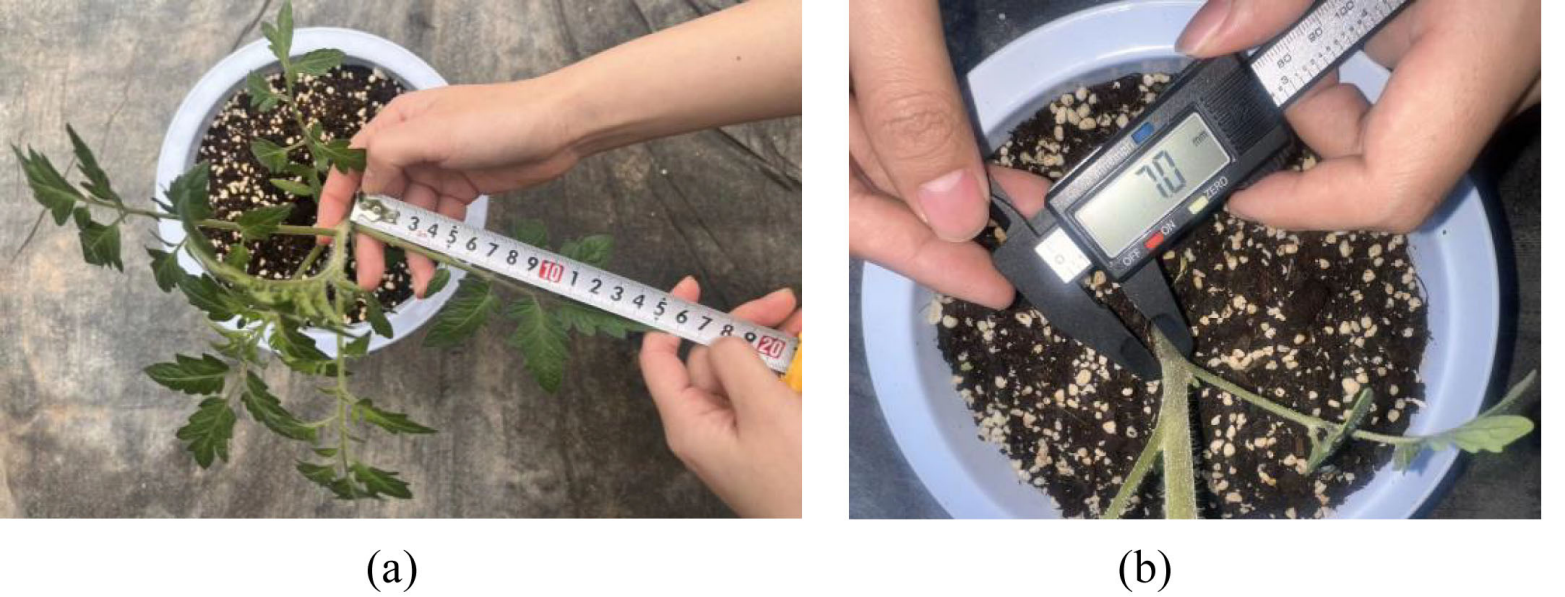
Diagram of manual measurement. **(a)** Measurement of leaf length. **(b)** Measurement of stem diameter.

For leaf length, we used a tape measure to measure along the main vein of the leaf from the base (the point where the petiole connects to the stem) to the tip of the leaf, as shown in **Figure 2a**. For pinnately compound leaves, we used the longest leaf axis length, including the terminal leaflet, as the standard. For stem diameter, to avoid irregular cross-sections of the stem caused by the plant’s phototropism, we uniformly selected a standard measurement point 1 cm above the soil surface, as shown in **Figure 2b**. We used a digital Vernier caliper for the “cross method” measurement. That is, at the midpoint of the internode, we measured the diameters of two mutually perpendicular directions and took their arithmetic mean as the reference value for the stem diameter of this plant.

### 2.2 Pre-processing of tomato canopy point cloud

#### 2.2.1 Removal of background point cloud

Raw point clouds contained substantial background and redundant information, which increased data volume and computational cost. To obtain a clear representation of the tomato canopy, background points were removed to retain only relevant plant structures through condition filtering. The spatial filtering thresholds were fixed according to the acquisition geometry described in Section 2.1. Specifically, points were retained within predefined XYZ ranges corresponding to the camera height (500–650 mm) and working distance (350–450 mm), ensuring a deterministic and reproducible background removal process. Points were retained if their attributes fell within the specified range, otherwise discarded (Wang et al., 2022). This paper selected the CloudCompare software (Borz and Proto, 2025) to remove background and pot parts from the original point cloud.

#### 2.2.2 Noise removal of canopy point cloud

After removing the background, noise information still exists around the plant. To obtain a more accurate point cloud of the tomato plant, noise removal is an important step. The noise of point clouds refers to invalid points collected by sensors. It can lead to false detections in target detection algorithms. Most 3D LiDAR scanners generate some level of noise during data acquisition (James et al., 2025). This noise can be caused by factors such as internal electronic components of the sensor, working temperature fluctuations, or interference between optical components. Additionally, external conditions like lighting, occlusions, and atmospheric conditions (e.g., fog, smoke, and dust) can also affect the captured PCD (Khajuria et al., 2025). For instance, in ToF sensors, each pixel has a physical size. When measuring object edges, a single pixel receives light reflected from both the foreground and background simultaneously. The combined energy results in multiple distances in the sensor’s raw data, causing flying pixels (Shahandashti et al., 2024).

**Figure 3:**
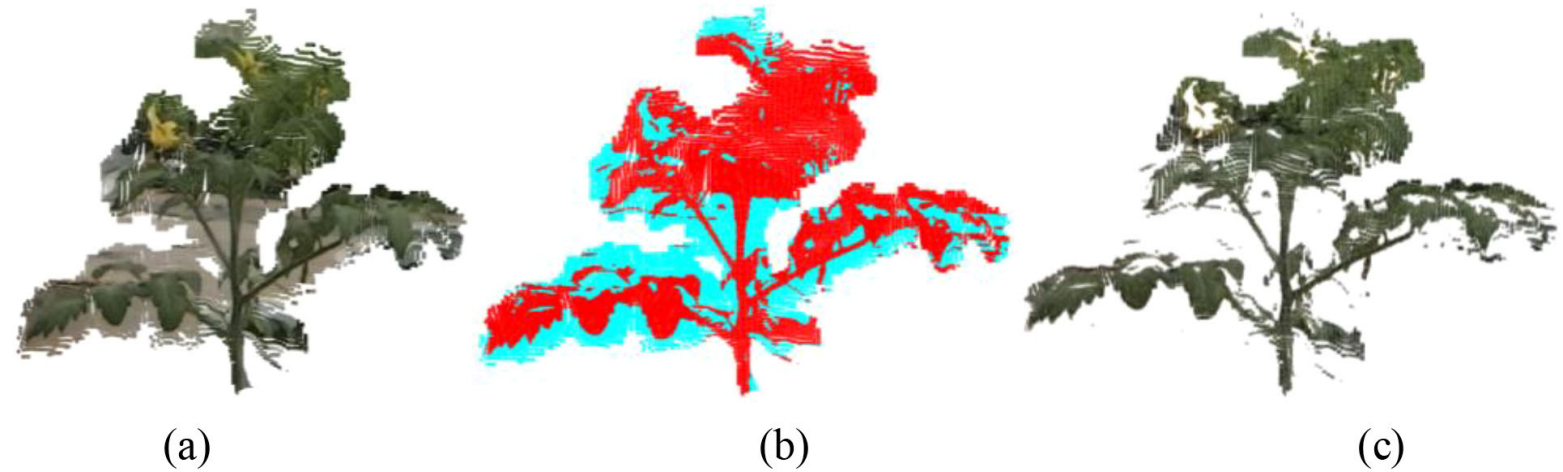
K-means clustering denoising. **(a)** Before tomato point cloud denoising. Canopy point clouds with flying pixels (original PCD after preprocessing), where the gray points in the figure are flying pixels, the green points are tomato plants, and the yellow points are flowers. **(b)** Color blocks after clustering. The point clouds were divided into two parts after clustering. The red part is tomato plants, and the blue part is flying pixels. **(c)** After clustering the tomato point cloud, most flying pixels were removed, and the whole plant was retained. PCD, point cloud data.

Lens scattering and crosstalk between pixels can also contribute to noise, sometimes causing significant background distortion. When laser point cloud scanning encounters certain special target surfaces, the excessive energy reflected from high-reflectivity surfaces can lead to a phenomenon known as “laser bloom”. Laser bloom refers to the expansion of a point cloud image around the laser-scanned high-reflectivity target, making the original object appear larger. This can occur due to the material properties and surface roughness (uneven surfaces altering the emission angle of point clouds) of the target object (Harcourt et al., 2024). Greenhouse environment, acquisition equipment, and surface characteristics of tomato plants contributed to the presence of flying pixels around point cloud data, as shown in **Figures 3a, b**.

From **Figures 3a, b**, these noises, although occupying only a small portion of memory resources, made the details of some parts of the tomato plant, such as stalks and leaves, become distorted. It affected the accuracy of model recognition and segmentation. However, these points were densely spaced and embedded in the overall plant point cloud. In fact, these points were not outliers. In **Figures 3a**, the colors of the flying point noise are visually or numerically significantly different from the colors of the tomatoes. These noise points will tend to form different clusters from the point cloud of tomato plants, and the situation can be regarded as an abstract “background-target” binary classification problem model. Under the condition of determining the number of classification clusters, the K-means algorithm can reasonably, quickly, and effectively segment the color clusters based on the color similarity so as to effectively separate the noisy points from the tomato point cloud. In this paper, we chose to use the K-means color clustering segmentation algorithm according to the characteristics of the tomato point cloud.

In this process, the RGB attributes of each point in the 3D point cloud were extracted and arranged as a color feature matrix for K-means clustering. The algorithm groups point with similar color distributions into *k* clusters, where each cluster represents a dominant color region within the scene. *k* was determined according to the number of major peaks observed in the RGB color histogram of each point cloud. When two dominant color distributions were present, *k* was set to 2. For samples exhibiting additional distinct color components, *k* was increased to 3 or 4 accordingly. This selection rule was applied consistently across all experiments. After clustering, the cluster corresponding to the predominant green vegetation was retained as the tomato plant region, as illustrated in **Figure 3c**.

**Figure 4:**
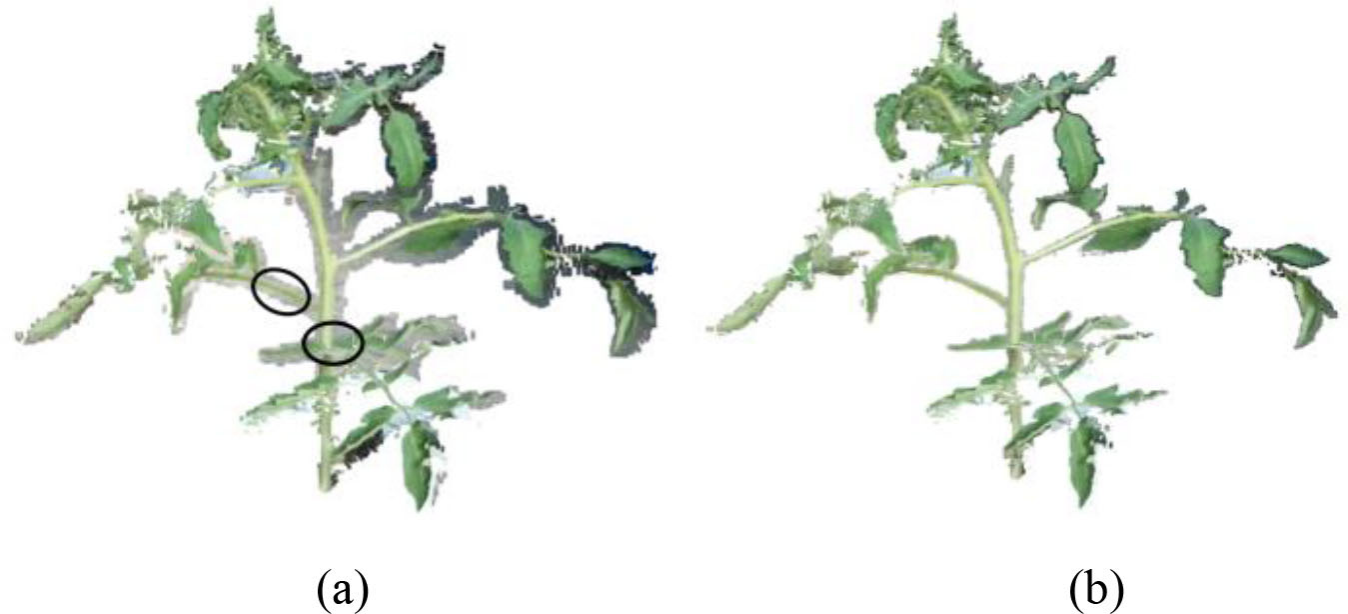
Segmentation results of EXG. **(a)** Point clouds of canopy with flying pixels. The black circles indicate the similarity between the color of flying pixels and the color of plants. **(b)** Segmentation results. The green part can preserve intact organs such as stems and leaves.

To achieve a more desirable clustering result, the characteristics of the point cloud should be considered when setting the number of clusters *k*. A smaller *k* may merge distinct color regions and cause information loss, while an excessively large *k* may lead to over-segmentation and fragmented clusters. During the data acquisition process, however, some flying pixels may have colors similar to stalks when the lighting is strong, as shown within the black circle in **Figure 4a**. When using the K-means algorithm, it is easy to filter out the tomato’s point cloud. In such cases, leveraging the super-green value feature can help distinguish between flying pixels and a tomato plant, thus achieving the segmentation.

The K-means algorithm performs well in clustering point cloud blocks with large color differences. For point cloud blocks with small color differences, this paper chose to use excess green value segmentation for clustering. Excess Green Index ( ExG). Segmentation is an image segmentation technique, primarily designed to separate green vegetation in an image. It relies on the dominance of green in plant leaves to distinguish vegetation from non-vegetation areas. Vegetation typically exhibits a higher green component in the RGB color space compared to red and blue. By calculating the ratio or difference between a pixel’s green component and the other color components, the algorithm determines whether the pixel belongs to a green vegetation region. The basic steps of the algorithm are as follows:

1. Color space transformation. Convert the color of each pixel from its original color space to a more suitable one for vegetation analysis.
2. Calculation of ExG. For each pixel in the point cloud, compute its ExG value using Equation 1 (Kanaley et al., 2025).

(1) ExG=2G−R−B

where R, G, and B represent the components of red, green, and blue, respectively. The values of R, G, and B range from 0 to 255. In this study, the threshold of ExG was dynamically determined for each sample based on the statistical distribution. Then, the histogram of ExG values was analyzed, and the threshold was selected near the valley point between vegetation and non-vegetation peaks to achieve optimal separation. The value 16 shown in **Figure 4b** corresponds only to that illustrative example and was not used as a fixed threshold across all experiments.

### 2.3 Dataset expansion and partitioning

Model training requires a large amount of point clouds. In this paper, tomato leaves were labelled using the CloudCompare software. Due to the complex interlacing of newborn leaves in tomato growing points, it was difficult to distinguish morphology and number of leaves by both visual identification and manual labelling (Jiang et al., 2025). For annotation, the canopy was divided into three semantic categories: stem, growing point, and leaves. The stem was labeled as “stem”, and its value was 0. The growing point was labelled as “canopy”, and the value was set to 1. Individual leaves were treated as separate instances; the value was set from 2 to n within each point cloud. To prevent data leakage, the dataset was divided at the level of original plant acquisitions prior to data augmentation. The dataset was split into training and testing subsets at a ratio of 7:3, resulting in 68 training samples and 29 testing samples.

**Figure 5:**
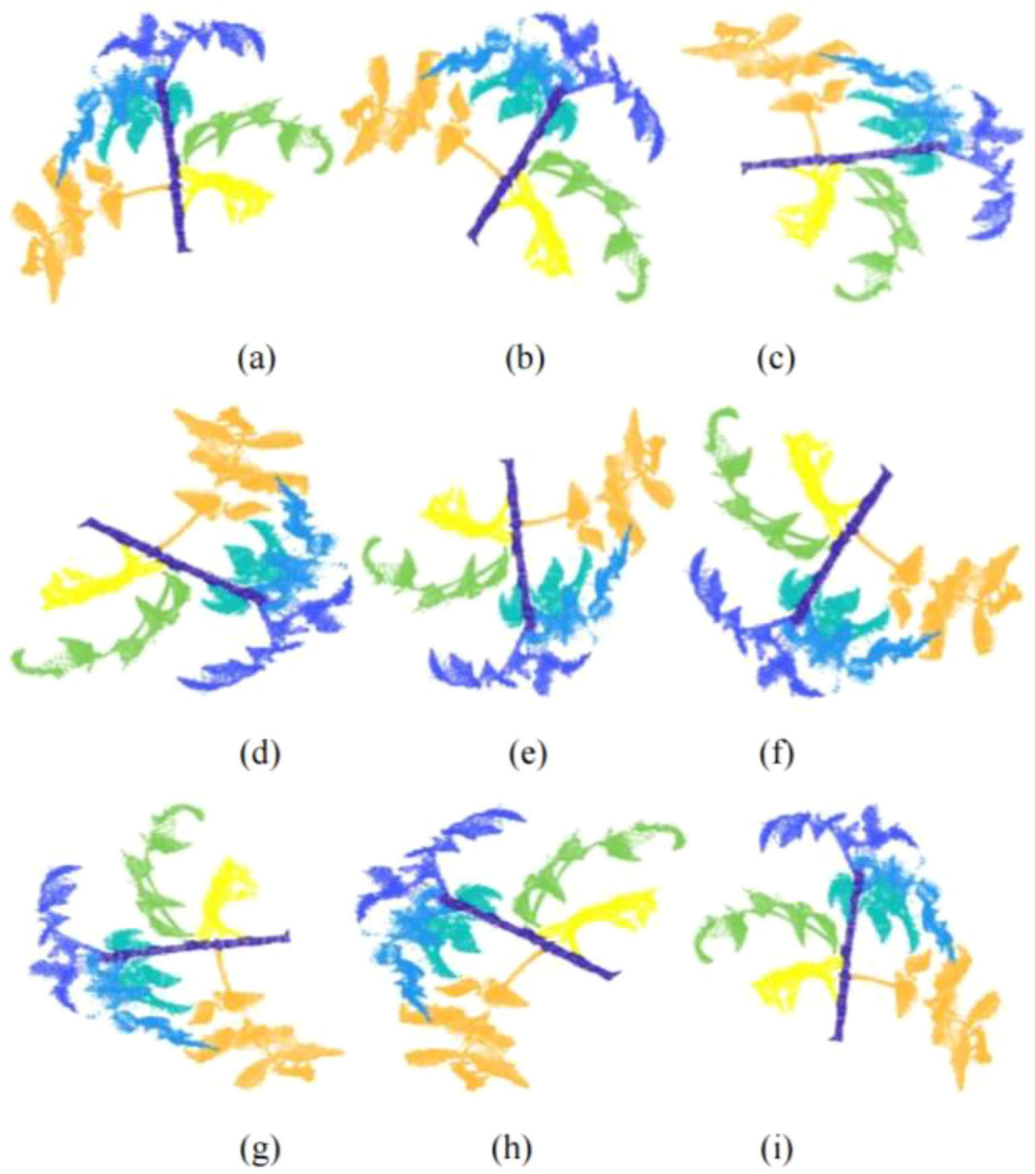
Illustration of data augmentation, where **(a)** denotes the raw sample. **(b–i)**, arranged from top to bottom and left to right, represent clockwise rotations of 45°, 90°, 135°, 180°, 225°, 270°, and 315°, as well as −180° (horizontal mirror), respectively.

Data augmentation was applied independently within each subset. Augmentation included coordinate inversion (x, y, z → −x, −y, −z), rotations from 45° to 315° at 45° intervals, and horizontal mirroring (−180°). As a result, each original sample was expanded to approximately 16 samples. After augmentation, the final TPCD contained 1,552 samples, including 1,088 training samples and 464 testing samples. In total, 5,008 leaf instances, 1,552 stems, and 1,552 growing points were annotated. The expanding results of the sample are shown in **Figure 5**.

### 2.4 A new VGDS-PointNet++

Due to the disordered and unstructured nature of point clouds, it was difficult to apply neural networks directly in segmentation tasks. PointNet can work directly on point cloud data and can make use of symmetric functions like maximum pooling. The sensitive problem of the order of input points is solved effectively. In PointNet model (Hasan et al., 2024), given an unordered set of points $\left\{x_{1},x_{2},\cdots x_{n}\right\}$ with $x_{i}\in R^{d}$, define a set function f:x→R. Map a set of points to a vector:

(2) $$f(x_{1},x_{2},\ldots ,x_{n})=\gamma \left(\underset{i=1,\ldots ,n}{\max}\left\{h(x_{i})\right\}\right)$$

where γ and h are multilayer perceptron networks.

The function f in Equation 2 keeps the input points’ arrangement and can approximate any continuous function. However, PointNet lacks a hierarchical feature learning module like a convolutional neural network, making it less effective at capturing subtle local structures and shape changes. This affects its segmentation and recognition accuracy in complex 3D environments. PointNet++ improves on PointNet by adding a hierarchical structure, enabling better handling of detailed features in complex regions and making it more adaptable to various point cloud scenarios (Ma et al., 2024b).

**Figure 6:**
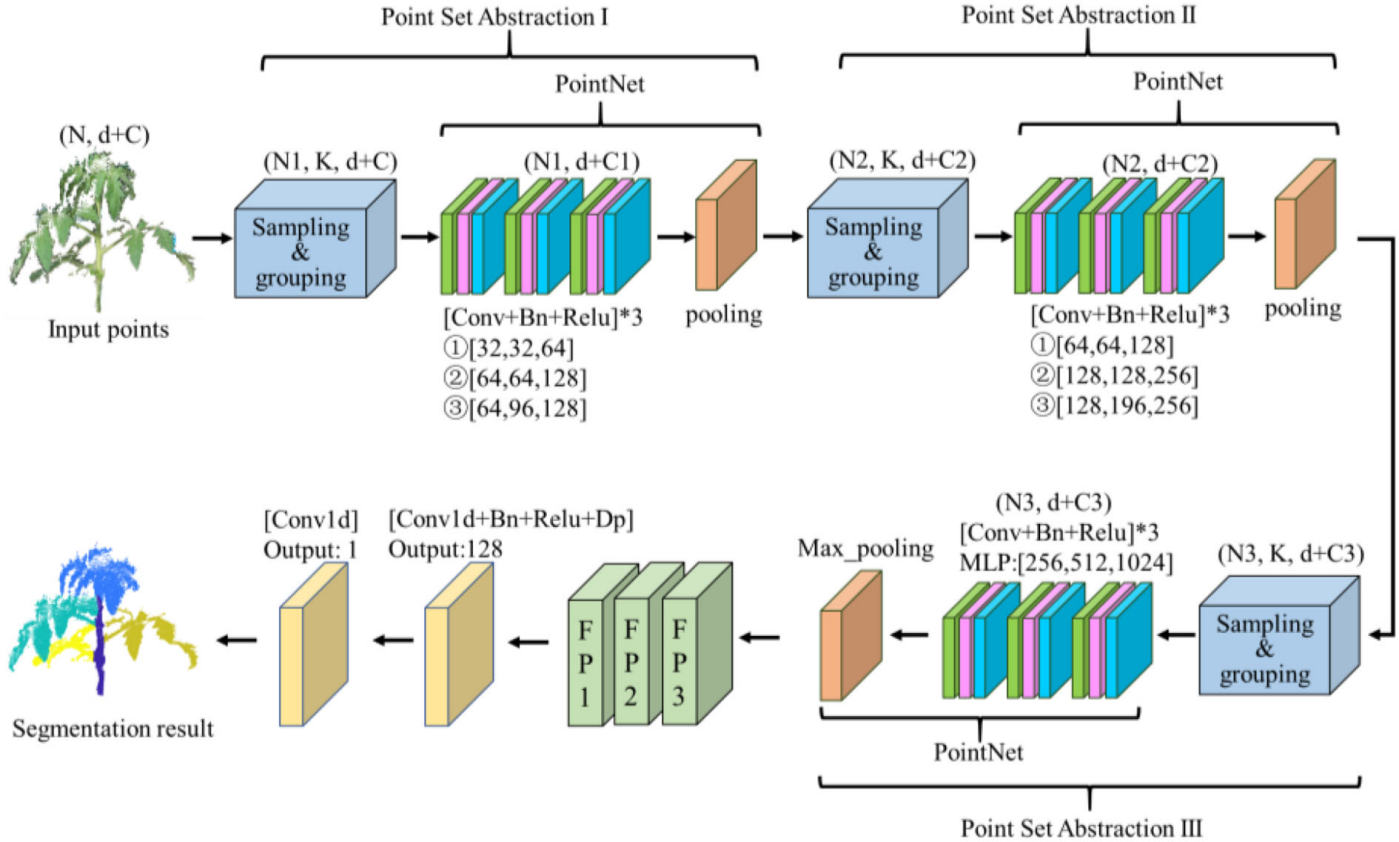
Architecture of PointNet++ (R and H, 2017b).

The PointNet++ model uses a multi-scale approach to capture local regions of different sizes. The network structure of PointNet++ is shown in **Figure 6**. It applies the principles of PointNet within each local region to extract point features, allowing the model to understand local features (Zhao et al., 2024). The PointNet++ model is composed of three SA levels. SA level is made of three key layers: sampling layer, grouping layer, and PointNet layer. The sampling layer selects a set of points from the input points, which defines the centroids of local regions. The grouping layer then constructs local region sets by finding “neighboring” points around the centroids. The PointNet layer uses a mini-PointNet to encode local region patterns into feature vectors (R and Y, 2017).

SA level takes an N×(d+C) matrix as input that is from N points with d-dim coordinates and C-dim point features. It outputs an $N^{\prime }\times (d+C^{\prime })$ matrix of N′ subsampled points with d-dim coordinates and new C′-dim feature vectors summarizing local context. This paper describes the layers included in the SA level in the following paragraphs.

Sampling layer. Given input points $\left\{x_{1},x_{2},\cdots x_{n}\right\}$, we used iterative farthest point sampling (FPS) to choose a subset of points $\left\{x_{i_{1}},x_{i_{2}},\cdots x_{i_{m}}\right\}$, such that $x_{i_{j}}$ is the most distant point (in metric distance) from the set $\left\{x_{i_{1}},x_{i_{2}},\cdots x_{i_{j-1}}\right\}$ with regard to the rest points. Given the same number of centers of mass, the entire point set can be better covered.

Grouping layer. The input to this layer is a point set of size N×(d+C) and the coordinates of a set of centroids of size $N^{\prime }\times d$. The output are groups of point sets of size $N^{\prime }\times K\times (d+C^{\prime })$, where each group corresponds to a local region and K is the number of points in the neighborhood of centroid points. Note that K varies across groups, but the succeeding PointNet layer is able to convert a flexible number of points into a fixed-length local region feature vector.

PointNet layer. In this layer, the input is $N^{\prime }$ local regions of points with data size $N^{\prime }\times K\times (d+C^{\prime })$. Each local region in the output is abstracted by its centroid and a local feature that encodes the centroid’s neighborhood. Output data size is $N^{\prime }\times (d+C^{\prime \prime })$. The coordinates of points in a local region are first translated into a local frame relative to the centroid point: $x_{i}^{\left(j\right)}=x_{i}^{\left(j\right)}-{\hat{x}}^{\left(j\right)}$ for i=1,2,…,K and j=1,2,…,d, where $\hat{x}$ is the coordinate of the centroid using PointNet as the basic building block for local pattern learning. Using relative coordinates together with point features, we can capture point-to-point relations in the local region.

In the PointNet++ model, FPS is used in the sampling layer. FPS is a point cloud downsampling technique commonly used for 3D object detection and segmentation. The core idea is to find the farthest point from the sampled point as the next sampling point. The sampling is as uniform as possible. After sampling, the general outline of the object can still be seen (Bahri et al., 2025). Furthest point downsampling ensures better coverage of the entire point cloud. However, the computational cost of FPS is high, especially for large-scale datasets, as each iteration needs to compute the farthest distance from all remaining points to the currently selected point set. The time complexity of FPS is usually related to the number of points and the number of sampling points selected and grows non-linearly with the size of the point cloud.

**Figure 7:**
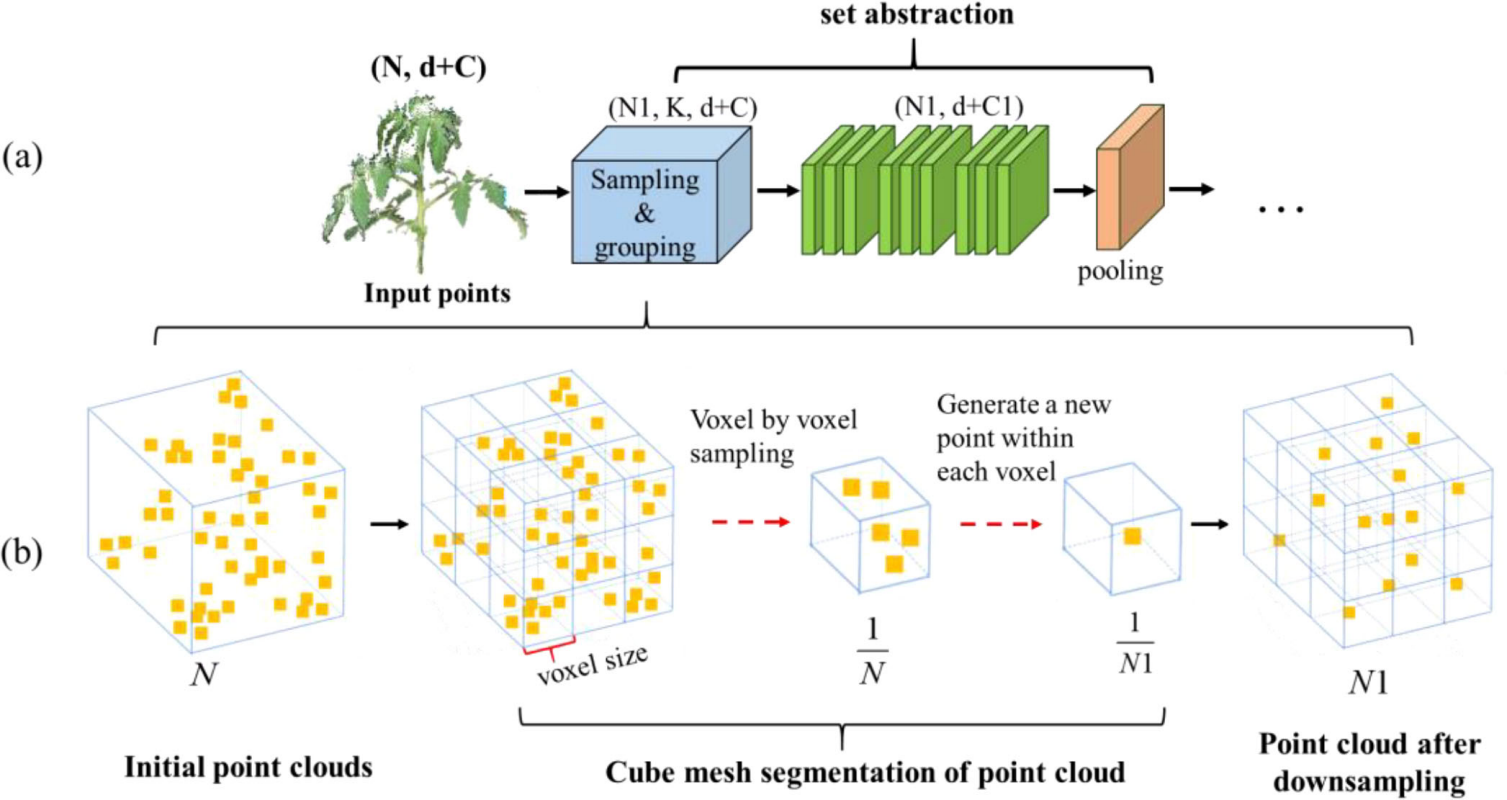
Set abstraction of VGDS-PointNet++. **(a)** PointNet++. The input part of PointNet++ and the first SA module. **(b)** Overview of voxel grid downsampling pipeline. VGDS, voxel grid downsampling; SA, Set Abstraction.

Voxel grid downsampling divides the entire 3D space covered by the point cloud according to a predefined voxel size with equal spacing, retaining only one point in each voxel, and preserving the overall structure and shape characteristics of the original data as much as possible. This reduces the number of points in the original point cloud, achieves data compression and noise reduction, and simplifies the subsequent point cloud analysis, modelling, and visualization processes (Huang et al., 2025). The core idea of voxel mesh downsampling is to replace the set of points within a voxel with a single point. This sampling point can be the center of mass of all points in the voxel; it can be the center point or the point closest to the center point. The process of voxel grid downsampling is illustrated in **Figure 7b**.

Voxel grid downsampling has lower time complexity compared to other algorithms, such as FPS, when dealing with large-scale datasets. The distribution of sampling points is more uniform and suppresses noise to some extent. The distance of sampling points can be indirectly controlled by setting the voxel size, that is, the sparseness of the sampled point cloud. Considering that the PointNet++ model is needed to handle a large number of tomato point clouds in this paper, on the basis of the original PointNet++ model, the sampling layer is optimized, and VGDS-PointNet++ is constructed. The improvement process is shown in **Figure 7**.

The Adam optimizer was adopted for model training due to its adaptive learning rate mechanism and stable convergence behavior. Adam combines momentum and adaptive gradient estimation to improve optimization efficiency during backpropagation (Krutikov et al., 2025). To mitigate potential overfitting, several regularization strategies were applied. First, weight decay (L2 regularization) was introduced with a decay rate of 0.0001. Second, dropout with a rate of 0.5 was incorporated in the fully connected layers to reduce co-adaptation of features. Third, early stopping was employed based on validation performance to prevent excessive training once convergence was achieved. In addition, data augmentation was performed prior to training to enhance sample diversity.

### 2.5 Calculation of leaf length

Calculating the blade length requires obtaining the complete skeleton of the blade. Common skeleton extraction algorithms, such as the Laplacian operator iteration algorithm, rely on a closed point cloud model. A closed point cloud model is a discrete data structure used to represent a 3D shape, which consists of a set of points, edges, and faces. This model is capable of capturing geometry and overall structure and has good geometric and topological properties (Xu et al., 2025). However, during data collection, due to the tomato planting pattern, single-view horizontal point clouds are easier to obtain and more efficient. However, single-view point clouds are non-closed. Therefore, it is necessary to improve the skeleton extraction strategy.

**Figure 8:**
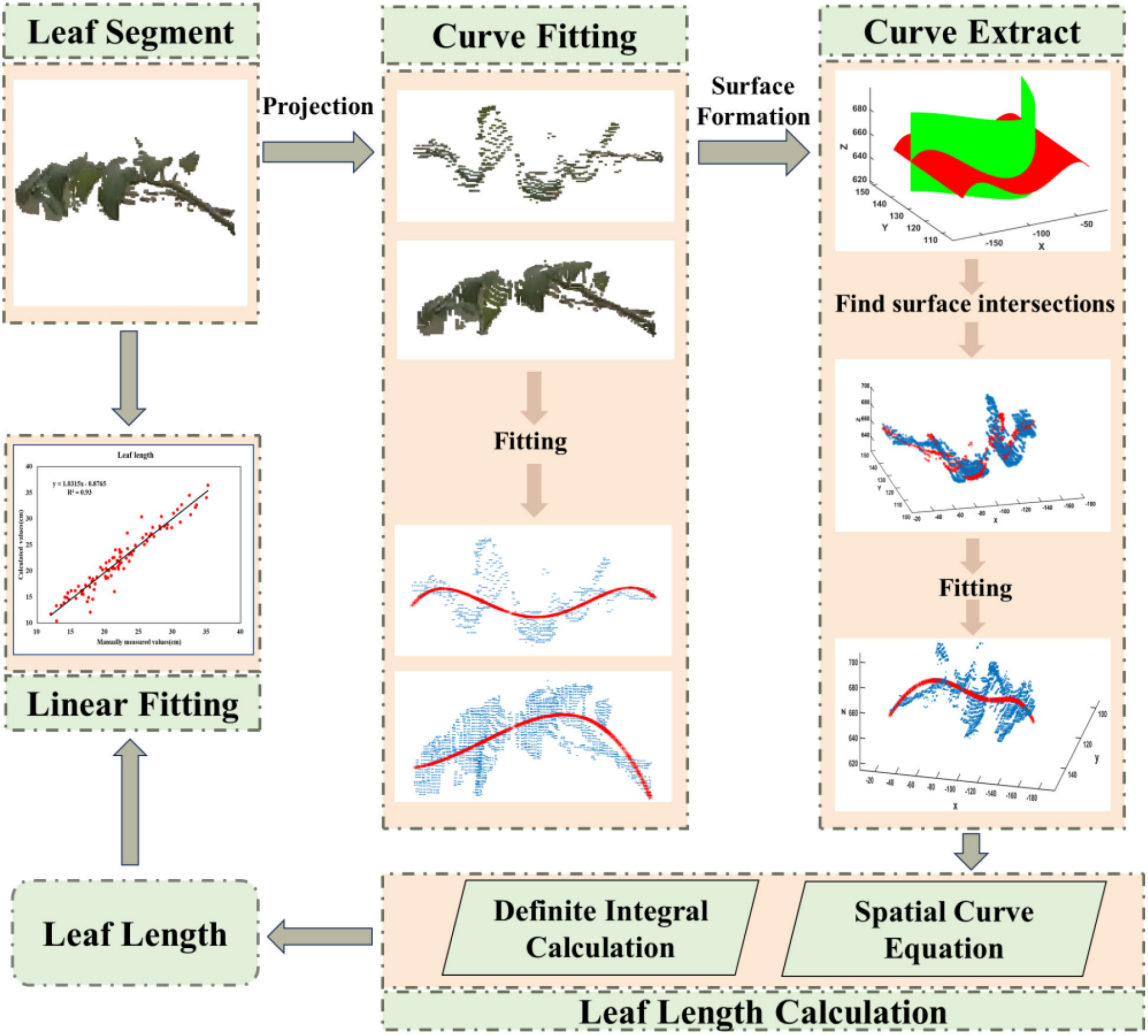
Process of fitting spatial curves.

Aiming at the limitations of the data collection environment and the problem of algorithm inapplicability, this article combines the principle of planar projection, the principle of slicing, and the curve fitting method and applies the least squares curve fitting to three-dimensional space to extract the spatial curves of the tomato leaf point cloud. The least squares method is a widely used mathematical method for data fitting, especially in curve fitting. It aims to find a curve (usually a mathematical model) such that the sum of the squares of perpendicular distances from all data points to this curve is minimized (Brugnano et al., 2024). The precise procedures shall be as follows. Tomato leaf point cloud *P* was first projected to the *XOY* and *YOZ* planes. The horizontal projections *Pxoy* and *Pyoz* of the leaf point cloud were obtained. Then, the horizontal projections *Pxoy* and *Pyoz* were fitted separately by combining the above equations. The fitted curves *Lxoy* and *Lyoz* were calculated, and the corresponding surfaces *Mxoy* and *Myoz* were constructed. Finally, the intersection line Lxyz of the two 3D surfaces was extracted as the 3D curve fitting result of leaf point cloud. The fitting process is shown in **Figure 8**.

Given a set of observed data points $\left(x_{i},y_{i}\right)$, i=1,2,3…,n, the sum of squares of the errors between the predicted and actual observations is minimized by optimizing model parameters. This approach assumes that the error in data (the difference between actual and predicted values) conforms to a Gaussian distribution. Therefore, the best fit is found by minimizing the sum of squares of the errors (Wu and Liu, 2024). The fitting steps are as follows:

(1) According to the characteristics of data, choose the appropriate curve model. This paper uses a non-linear polynomial model. As shown in Equation 3.

(3) $$y=f(x)=a_{0}+a_{1}x+a_{2}x^{2}+\cdots +a_{m}x^{m}$$

where $a_{0},a_{1},\ldots ,a_{m}$ are model parameters.

(2) Construct an objective function, which is the sum-of-squares-of-errors function. The objective is to minimize the total sum of squares of the errors from all data points to model predictions. As shown in Equation 4.

(4) $$S=\sum_{i=1}^{n}{\left[y_{i}-f(x_{i})\right]}^{2}$$

where $f\left(x_{i}\right)$ is the predicted value at $x_{i}$ given by model.

(3) Solve for the partial derivatives of error function s with respect to each parameter in order to compute the minimized sum of squares of errors. As shown in Equation 5.

(5) $$\frac{\partial S}{\partial a_{j}}=-2\sum_{i=1}^{n}(y_{i}-f(x_{i}))\frac{\partial f(x_{i})}{\partial a_{j}}=0$$

(4) Based on the above steps, the best-fitting parameters are obtained. For linear problems, fitting parameters can be directly used in matrix operations. For non-linear problems, numerical optimization is required before operations.

In three-dimensional space, combined with the principle of plane projection, the leaf point cloud is projected onto two specific planes in space. Combined with the above formula, the two horizontally projected plane lines are fitted, surfaces are constructed, and the intersection line of the two surfaces is taken as the 3D curve fitting result of the leaf point cloud. The length of the curve is the length of the leaf.

The length of a space curve can be calculated by integration. Calculating the length of a space curve involves integrating the lengths of infinitesimal line segments on the curve. This calculation is based on the arc length equation in calculus. The slope of the curve at each point is obtained by solving the derivatives of the parametric equations. In turn, the length of the curve is calculated (Calissano et al., 2024) if there is a spatial curve defined over a parameter t, denoted r(t)=(x(t),y(t),z(t)), where t varies over the interval [a,b]. Then, the length of this curve from point t=a to t=b can be calculated by the following Equation 6:

(6) $$L=\int_{a}^{b}\sqrt{{\left(\frac{dx}{dt}\right)}^{2}+{\left(\frac{dy}{dt}\right)}^{2}+{\left(\frac{dz}{dt}\right)}^{2}}dt$$

where $\frac{dx}{dt}$, $\frac{dy}{dt}$, $\frac{dz}{dt}$ represent the rate of change of curve in each direction.

### 2.6 Calculation of stem diameter

In addition to leaf length, stem diameter is also a key indicator of plant health and growth, reflecting the plant’s ability to accumulate biomass, store nutrients, and maintain structural integrity to prevent lodging. During the seedling stage of tomatoes, the stem diameter is not uniform; therefore, a fixed position is required for accurate measurement. In this experiment, the stem diameter measurement position was set at 1 cm above the base of the plant.

After obtaining the stem segmented by the model, project the point cloud onto the XOY or XOZ planes according to the camera coordinates to ensure the front profile of the stem. Then, slice the projected result to extract the point cloud in the area close to the manually measured position. Construct an oriented bounding box (OBB) for the point cloud in this area, and obtain its rotation matrix. The construction of the OBB is achieved by minimizing the volume of the bounding box, with its spatial orientation described by a rotation matrix R (3 × 3), which satisfies the orthogonality condition $R^{T}R=I$ (where I is the 3 × 3 identity matrix). Adjust the point coordinates $P_{roi}$ in the sliced point cloud using the rotation matrix to obtain the rotated point cloud ${P^{\prime }}_{roi}=\{{p^{\prime }}_{j}|{p^{\prime }}_{j}=R\cdot p_{j}\}$, where $p_{j}\in P_{roi}$. Align the main direction of the adjusted point cloud with the coordinate system axes, laying the foundation for subsequent projection calculations. Finally, calculate the stem diameter through projection and ring fitting. Project the rotated point cloud ${P^{\prime }}_{roi}$ onto the XOY plane to obtain the two-dimensional point set $Q=\{q_{k}|q_{k}=({x^{\prime }}_{k},{y^{\prime }}_{k}),k=1,2,\ldots ,M\}$. Using the least squares method to perform annular fitting on the 2D point set Q, the annular equation is as follows (Equation 7):

(7) $${\left(x-a\right)}^{2}+{\left(y-b\right)}^{2}=r^{2}$$

where (a,b) is the center coordinate of the ring and r is the radius of the ring.

**Figure 9:**
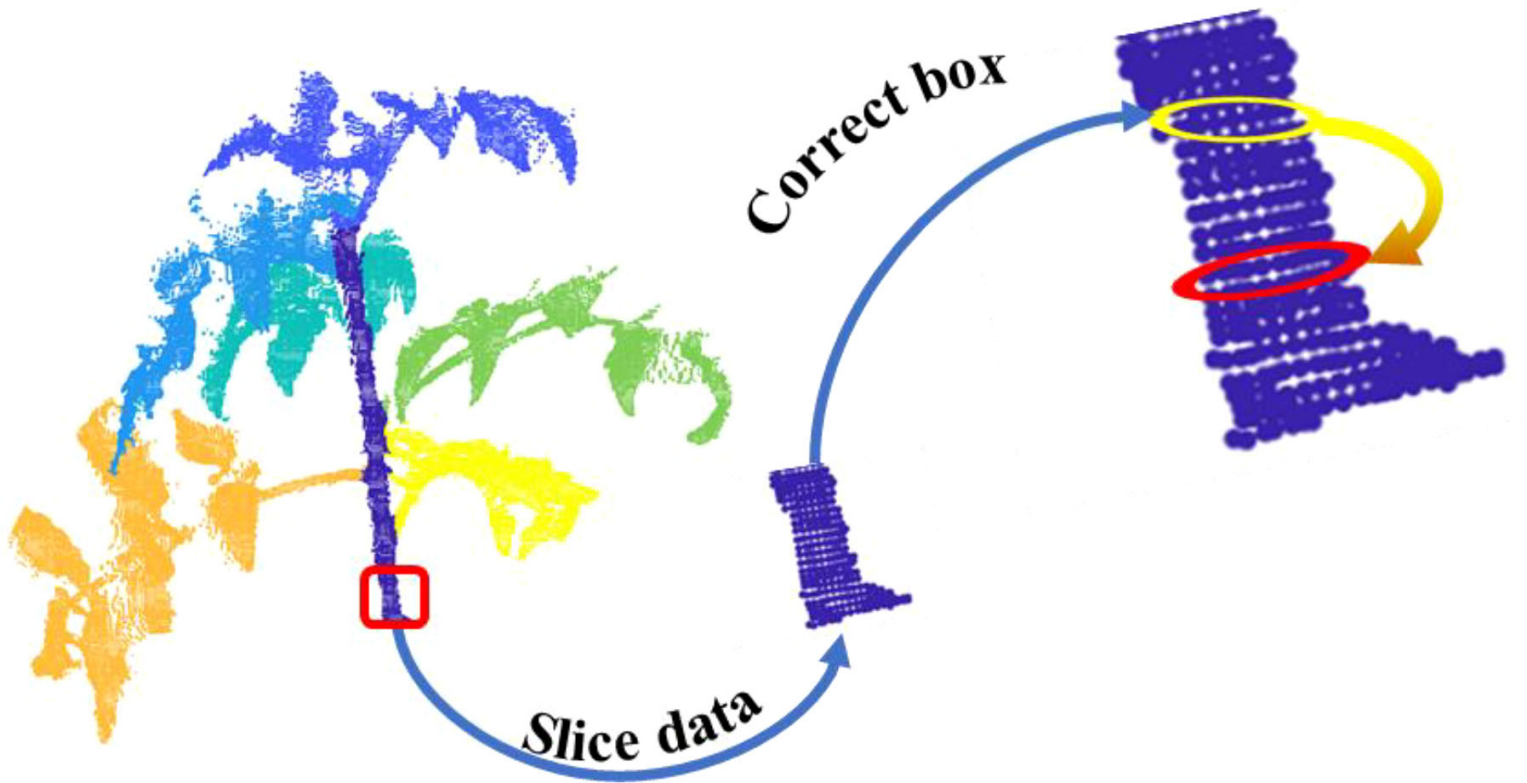
Calculation process of stem diameter.

During the fitting process, the optimal parameters a,b,r are obtained by minimizing the objective function $\sum_{k=1}^{M}{\left[{\left({x^{\prime }}_{k}-a\right)}^{2}+{\left({y^{\prime }}_{k}-b\right)}^{2}-r^{2}\right]}^{2}$. The stem thickness d is determined by the diameter of the ring, d=2r. The calculation process is shown in **Figure 9**.

## 3 Results and discussions

All reported metrics [Acc, Precision, Recall, F1-score, and Intersection over Union (IoU)] were computed under this four-label setting, following relevant segmentation evaluation protocols. The evaluation metrics were defined as follows (Equations 8–12):

(8) $${\text{Accuracy}}_{k}=\frac{\sum_{k=0}^{2}TP_{k}}{N}$$

(9) $${\text{IoU}}_{k}=\frac{TP_{k}}{TP_{k}+FP_{k}+FN_{k}}$$

(10) $${\text{Precision}}_{k}=\frac{TP_{k}}{TP_{k}+FP_{k}}$$

(11) $${\text{Recall}}_{k}=\frac{TP_{k}}{TP_{k}+FN_{k}}$$

(12) $${F1}_{k}=\frac{2\cdot {\text{Precision}}_{k}\cdot {\text{Recall}}_{k}}{{\text{Precision}}_{k}+{\text{Recall}}_{k}}$$

where N denotes the total number of points, and k∈{0,1,2} denotes each label. Metrics were first calculated for each label. The final metrics reported values in **Tables 1**, **2** were computed as support-weighted averages across all labels to account for class imbalance. The weight for each label was determined by the proportion of points belonging to that label in the dataset. As shown in Equation 13:

**Table 1:** Training results for the tomato dataset on each model.

Model	Training set		Testing set	
	Accuracy	mIoU	Accuracy	mIoU
Point Transformer v1	71.42%	60.84%	68.81%	55.83%
Point Transformer v3	96.69%	89.57%	95.86%	86.91%
Point-BERT	95.96%	88.33%	90.78%	79.24%
PointNet	76.46%	64.96%	72.69%	43.14%
PointNet++	96.06%	86.31%	95.52%	84.52%
**VGDS-PointNet++**	**97.17%**	**90.77%**	**96.57%**	**87.59%**

The bold values represent the results of VGDS-PointNet++, which is also the best model among the results in the table. mIoU, mean Intersection over Union; VGDS, voxel grid downsampling.

**Table 2:** Segmentation results of each label.

Indicators Labels	Accuracy	Precision	Recall	F1	mIoU
Stem	98.93%	89.69%	86.35%	87.66%	79.12%
Growing point	94.11%	92.57%	93.17%	92.87%	91.69%
Leaf	92.12%	95.86%	93.70%	93.21%	93.77%

mIoU, mean Intersection over Union.

(13) $$w_{k}=\frac{N_{k}}{\sum_{j=0}^{2}N_{j}}$$

where $N_{k}$ denotes the number of points labelled as *k*. The final metric was then calculated as followed in Equation 14:

(14) $${\text{Metric}}_{\text{weighted}}=\sum_{k=0}^{2}w_{k}\cdot {\text{Metric}}_{k}$$

Through these revisions, the baseline comparison has been revalidated, clarified, and strengthened. We greatly appreciate the reviewer’s careful and constructive comments, which have helped us substantially improve the rigor and transparency of the experimental evaluation.

### 3.1 Algorithmic complexity analysis of sampling methods

To examine the intrinsic computational complexity of different sampling strategies, a dedicated CPU-only benchmark was conducted to compare voxel grid downsampling with standard FPS. This analysis was performed independently from the subsequent segmentation training experiments and focused exclusively on algorithmic runtime under identical hardware conditions. All procedures were implemented in MATLAB and executed without GPU acceleration to ensure a consistent execution pathway.

After background and noise removal, each raw point cloud contained approximately 2 million points. These data were downsampled to approximately 100,000 points before training. For a fair comparison, FPS was configured to generate the same number of sampled points as produced by voxel grid downsampling (Sugimoto et al., 2025). Two voxel grid sizes (1 and 1.5 mm) were evaluated, consistent with the millimeter coordinate unit of the sensor data. Runtime was measured per PLY file across 97 point clouds. **Tables 1**, **3** present the corresponding results.

**Table 3:** CPU-only runtime comparison per PLY file.

Method	Grid size (mm)	Input points	Output points	Runtime (s)
Voxel	1	~2,000,000	~100,000	0.102 ± 0.135
Voxel	1.5	~2,000,000	~100,000	0.206 ± 0.152
FPS	–	~2,000,000	~100,000	8,493 ± 5,191

FPS, farthest point sampling.

As shown in **Table 3**, under identical CPU-only conditions and with matched output point counts (~100,000 points), voxel grid downsampling required substantially less processing time than standard FPS when operating on multi-million-point inputs (~2,000,000 points). The average runtime of voxel grid downsampling was 0.102 s (1 mm) and 0.206 s (1.5 mm), whereas FPS required 8,493 s on average. The large difference primarily stems from the underlying computational mechanisms. Voxel grid downsampling partitions the space into regular grids and aggregates points within each voxel, leading to near-linear complexity with respect to input size. In contrast, standard FPS iteratively updates distances between all input points and selected centroids, resulting in significantly higher computational cost when both input size and sampled size are large.

Although the benchmark evaluates sampling complexity under CPU-only conditions, its implications extend to the segmentation pipeline. In practical training workflows, point clouds are first processed on the CPU and subsequently transferred to the GPU for model execution. Therefore, reducing preprocessing time directly lowers data handling overhead before GPU acceleration begins. In addition, sampling operations are repeatedly invoked inside the SA modules during training. In the original PointNet++ architecture, FPS is executed iteratively within each forward pass. In this study, FPS in the model was replaced with a GPU-based voxel sampling implementation, thereby reducing iterative distance computations during feature abstraction. Consequently, improvements observed in the preprocessing benchmark reflect the intrinsic computational advantage of voxel-based spatial partitioning over iterative FPS. While segmentation training is GPU-accelerated, minimizing sampling complexity contributes to improved scalability and more stable runtime behavior when handling large-scale point clouds.

### 3.2 Performance analysis of VGDS-PointNet++ model

All segmentation experiments were conducted on a workstation running Ubuntu 20.04. The hardware configuration included an Intel Xeon E5–2690 V4 CPU and an NVIDIA GeForce RTX 3080 Ti GPU with 12 GB of memory for training acceleration. The software environment consisted of Python 3.7 and TensorFlow 2.10.0. CUDA 11.4.0 and cuDNN 8.2.4 were used for GPU acceleration. To avoid out-of-memory errors during training, all models were trained under identical experimental settings: batch size = 7, optimizer = Adam, initial learning rate = 0.0001, weight decay = 0.0001, learning rate decay factor = 0.5, dropout = 0.5, number of input points = 20,000, and total training epochs = 100. The same train/test split and augmentation strategy were applied across all models. Early stopping was applied based on validation mean Intersection over Union (mIoU) to prevent overfitting.

**Figure 10:**
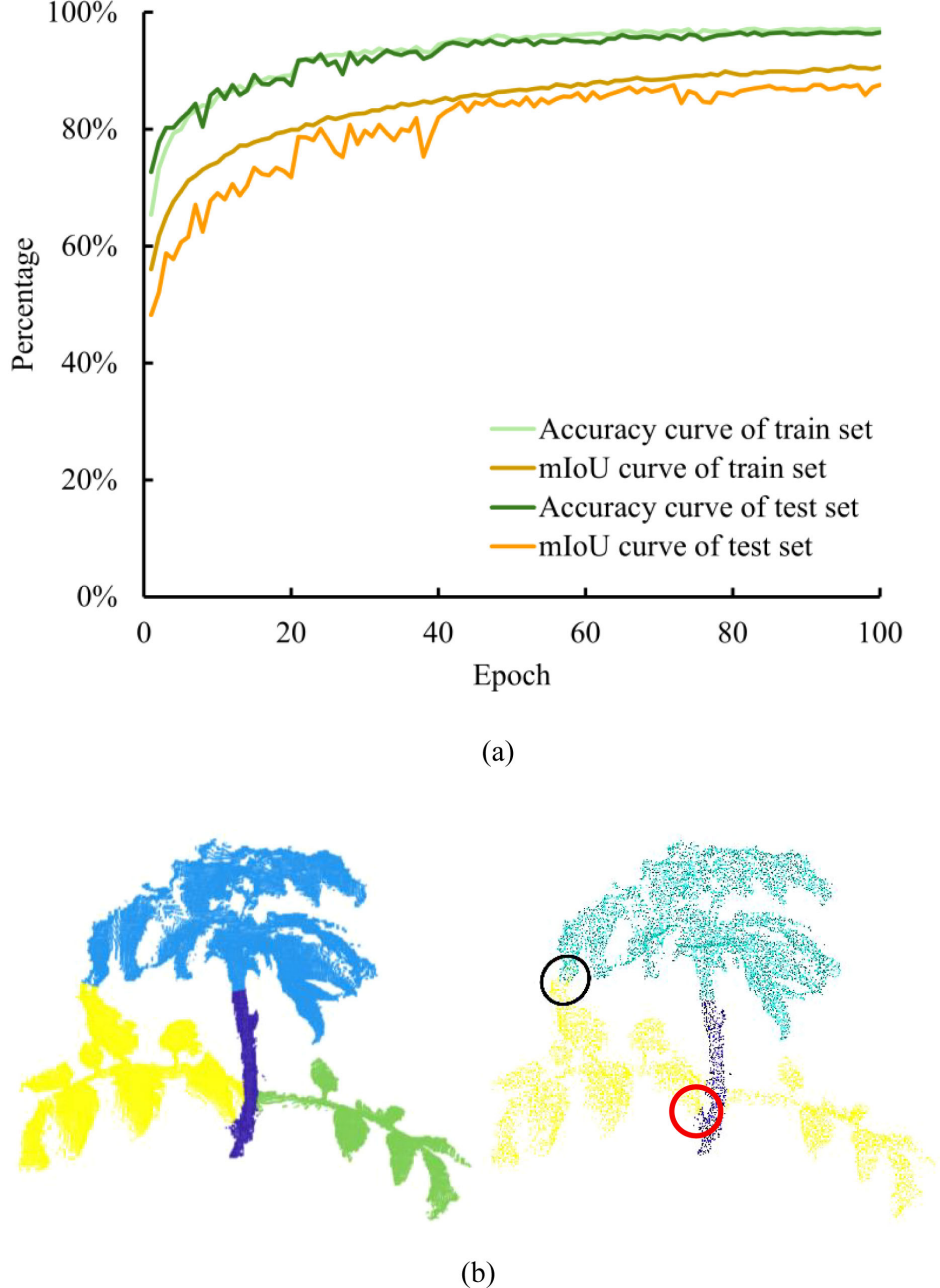
Training and segmentation results of VGDS-PointNet++. **(a)** Convergence process of Accuracy and mIoU for VGDS-PointNet++. **(b)** Comparison of point clouds before and after model segmentation. The black circles mark the part of the point cloud where the model segmented incorrectly between leaf and growing point. The red circles mark the part of the point cloud where the model segmented incorrectly between stem and leaf petiole. VGDS, voxel grid downsampling; mIoU, mean Intersection over Union.

The tomato dataset was evaluated on six models: PointNet, PointNet++, VGDS-PointNet++, Point Transformer v1, Point Transformer v3, and Point-BERT. Performance was evaluated in terms of Accuracy and mIoU, as illustrated in **Figure 10a**, following the standard definitions reported in the literature. The comparative results on the training and testing sets are summarized in **Tables 1**, **2**. Metrics were computed following established semantic segmentation evaluation protocols (Park et al., 2022; Liu et al., 2026; Yao et al., 2026).

The comparative results in **Table 1** demonstrate that all models were trained under identical conditions and achieved stable convergence on the tomato dataset. Classical models such as PointNet and Point Transformer v1 exhibited moderate performance, with larger gaps between training and testing results, indicating limited feature representation capacity for complex plant structures. In contrast, more advanced architectures, including PointNet++, Point Transformer v3, and Point-BERT, showed substantial improvements in both Accuracy and mIoU on the testing set. VGDS-PointNet++ achieved a testing Accuracy of 96.57% and an mIoU of 87.59%, which is comparable to or slightly higher than other high-performing models. These results indicate that integrating voxel grid downsampling into the hierarchical feature extraction framework does not degrade segmentation accuracy while maintaining stable generalization performance. Overall Accuracy and mIoU provided a global evaluation of segmentation quality, but the distribution of point instances across organ categories was not uniform. In the constructed dataset, leaf points accounted for approximately 60%, followed by growing points, while stem points represented the smallest proportion. Under such class imbalance conditions, aggregated metrics may be influenced more strongly by dominant categories. Therefore, a detailed per-class performance analysis is presented in **Table 2** to further examine error distribution and organ-level segmentation characteristics.

**Table 2** further presents the per-class segmentation performance and enables a more detailed examination of error characteristics. The leaf category achieved high precision (95.86%) and recall (93.70%), indicating both low false-positive and low false-negative rates, despite representing the largest proportion of points in the dataset. The growing point also demonstrated balanced precision and recall (92.57% and 93.17%), suggesting stable discrimination with limited systematic bias. In contrast, the stem category exhibited lower recall (86.35%) and mIoU (79.12%), indicating that most misclassifications occurred between the stem and leaf categories, particularly at the boundary. This higher false-negative tendency can be attributed to the geometric similarity and spatial continuity between stems and leaf petioles, particularly near boundary regions. Meanwhile, the relatively high stem precision (89.69%) suggests that excessive false positives were limited. Overall, the per-class metrics indicate that segmentation errors primarily arise from local structural ambiguity rather than dominant-class bias, and the model maintains reasonably balanced performance across organ types under class-imbalanced conditions.

As shown in **Figure 10b**, VGDS-PointNet++ achieved clear organ-level segmentation of leaves, growing points, and stems. The black circles highlight regions where misclassification occurred between the leaf and growing point categories. These errors were primarily located near transitional regions where the morphological boundaries between the growing point and emerging leaves were less distinct. The red circles indicate misclassification between the stem and leaf petiole regions. This observation is consistent with the per-class evaluation in **Table 2**, where the stem category exhibited lower recall compared to leaf and growing point classes, suggesting a higher false-negative tendency for stem points near boundary areas. These misclassifications were mainly concentrated at local organ junctions rather than across entire structures, indicating that the model effectively captured global organ geometry while exhibiting limitations in boundary discrimination. Despite these localized ambiguities, the overall segmentation performance remained stable, as reflected by the high class-wise precision and mIoU values reported in **Table 2**.

### 3.3 Discussion of point cloud segmentation result

The dataset comprised tomato seedlings that had not been subjected to vine-hanging treatment. In the greenhouse, contemporary agricultural practices predominantly employ methods such as substrate cultivation and hydroponics for ridge planting (Wang et al., 2024). Ridge planting, however, results in closer spacing between tomato plants and a more intricate surrounding environment. During the flowering and fruit-setting stages, plants are prone to shading each other, resulting in substantial leaf overlap. The segmentation of large overlapping leaves presents a significant challenge in the extraction of plant phenotypic traits from 3D point clouds. To address this, researchers have endeavored to enhance the performance of point cloud segmentation models by introducing various modifications to their components. In this study, the FPS strategy within the SA module of PointNet++ was replaced with voxel downsampling, facilitating faster and more accurate extraction of tomato leaf morphological characteristics. Incorporation of a hierarchical structure into PointNet++ improved the efficiency of the sampling layer. The grouping layer partitioned points within the target region into local regions, within which PointNet extracted salient features. Nonetheless, PointNet’s global awareness mechanism, characterized by a max-pooling layer, may compromise the detection of local features.

As depicted in **Table 2**, the VGDS-PointNet++ model effectively segmented points from different instances. It demonstrated efficacy in tomato segmentation tasks. Although leaf segmentation accuracy was marginally lower than Point Transformer v3, the model achieved high accuracy in segmenting growing points and stems. The model’s ability to capture leaf details could be improved by integrating multimodal features, such as leaf color and contours, into the input data. Moreover, optimizing local feature extraction and multimodal feature fusion techniques could further refine the model’s performance. Regarding the dataset, even though data augmentation was used to expand the dataset before training, it remains challenging for the dataset to cover all greenhouse scenarios. Factors such as weather, lighting conditions, and occlusions may affect the final experimental results.

The proposed method has several limitations worth noting. First, its performance is contingent upon a specific tomato dataset, which may diminish its accuracy when applied to other plant species. Second, despite utilizing sophisticated algorithms and models, the method may fail to capture all detailed features in complex scenes, potentially leading to classification errors in semantic segmentation tasks. Additionally, the accuracy of the data acquisition equipment is a critical factor; the quality of captured data is closely tied to the device specifications, which can influence segmentation accuracy. We will address these shortcomings and limitations in future work.

### 3.4 Results of tomato leaf length calculation

**Figure 11:**
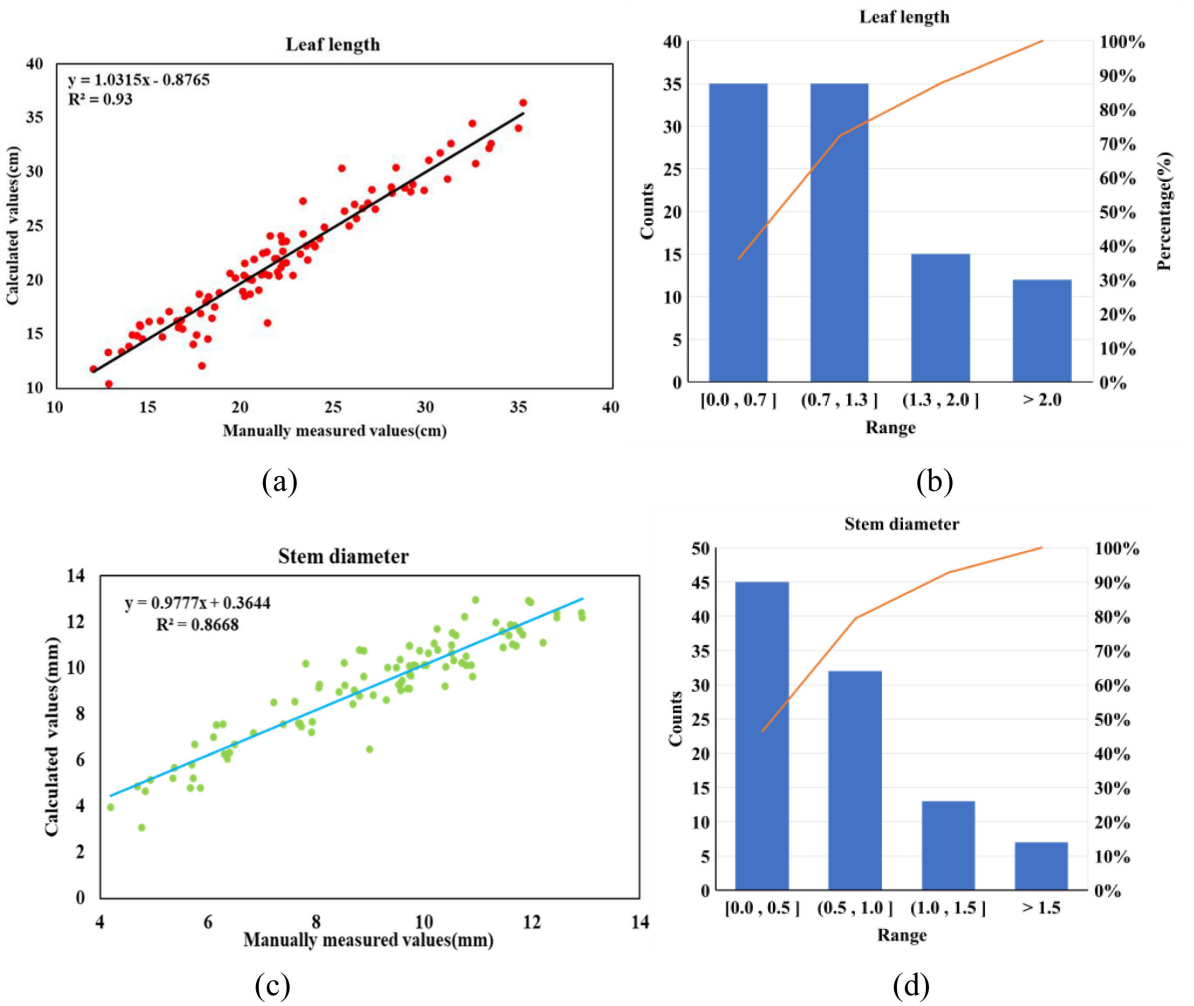
Analysis of extraction results of leaf length and stem diameter. **(a)** Linear correlation demonstration of leaf length. **(b)** Bias distribution of leaf length. **(c)** Linear correlation demonstration of stem diameter. **(d)** Bias distribution of stem diameter. In panels b and d, the blue bars represent the distribution of errors across different segment values, while the orange line represents the proportion of errors.

After segmenting, the leaf length and stem diameter of 97 samples were calculated. The relationship between the calculated values and the measured values is shown in **Figure 11**.

As shown in **Figures 11a**, the predicted leaf lengths exhibited a strong linear relationship with the manually measured values (*R*^2^ = 0.93), indicating that the proposed spatial curve fitting method can approximate leaf geometry under single-view acquisition conditions. However, correlation alone does not fully reflect prediction reliability. Therefore, the error distribution is further analyzed in **Figure 11b**. The absolute error of leaf length ranged from 0.02 to 5.73 cm, with a mean absolute error of 1.16 cm. Most samples were concentrated within low-error intervals, while a limited number of outliers contributed to the upper error range. These larger deviations were mainly associated with leaves exhibiting pronounced curvature, partial self-occlusion, or reduced point density after segmentation. Since the curve fitting relies on projected structural continuity, incomplete geometric information may propagate into length estimation errors.

For stem diameter estimation, **Figure 11c** shows a strong linear relationship between predicted and measured values (*R*^2^ = 0.8668). The measured stem diameters ranged from 4.20 to 12.94 mm. The maximum absolute error was 2.52 mm, and the minimum was 0.01 mm. The root mean square error (RMSE) was 0.7972 mm, and the mean absolute percentage error (MAPE) was 7.725%. The error histogram in **Figure 11d** indicates that most stem diameter errors were concentrated within small deviation intervals, while a few samples exhibited larger discrepancies. These deviations are primarily attributed to irregular or elliptical stem cross-sections, slight misalignment during slicing and projection, and local variations in point cloud density near the measurement region. Overall, the combined regression and error distribution analyses suggest that the proposed framework provides stable organ-level measurements under the current experimental conditions, while acknowledging the influence of structural complexity and data acquisition limitations on estimation accuracy.

### 3.5 Discussion of plant phenotype extraction

Single-view point cloud acquisition cannot fully capture the complete curvature and three-dimensional structure of tomato leaves. Variations in illumination, minor plant movement, and natural surface irregularities may affect data completeness and introduce measurement uncertainty. In cases where partial structural information is missing, skeleton extraction may produce geometrical deviations. To mitigate this limitation, the proposed method integrates planar projection and spatial curve fitting to obtain a stable approximation of leaf morphology for length estimation.

Leaf curling caused by water stress, excessive radiation, or disease can further influence fitting accuracy. Although robust statistical fitting was applied, polynomial models may not completely describe highly non-linear or severely deformed leaf structures. Similarly, stem diameter estimation is influenced by biological variability and measurement conditions. Tomato stems are not perfect cylinders and may exhibit ellipticity or irregular cross-sections. In addition, periodic physiological fluctuations during the day–night cycle can introduce temporal variability. These factors explain part of the observed deviation between predicted and manual measurements.

Despite these limitations, the proposed organ-level phenotyping framework has practical implications for precision greenhouse management. Leaf length and stem diameter can serve as quantitative indicators for seedling vigor screening, growth monitoring, and early stress detection. By establishing reference thresholds or ranking seedlings according to organ traits, greenhouse managers can perform automated grading, identify weak individuals, and adjust irrigation or environmental control strategies. Growing point segmentation further enables structural integrity assessment at early developmental stages.

For real-world deployment, stable sensor placement above seedling beds or conveyor systems is required to ensure consistent acquisition geometry. The pipeline must operate under standardized lighting conditions and achieve sufficient throughput for batch analysis. While robustness to occlusion and environmental variability remains a challenge, the proposed framework provides a technical foundation for integrating 3D perception with data-driven greenhouse decision support systems.

In terms of expanding the application scope, first, from the perspective of plant morphology, the core design of this model does not rely on specific color or texture features of tomatoes but focuses on modeling the topological structures and geometric relationships of stems and leaves. For example, although cucumbers and peppers have different fruit shapes, their pendant structures under trellising or vine training cultivation, internode distribution, and fruit-stem connection patterns exhibit high similarity to tomatoes. Therefore, this model is expected to be transferred to organ recognition and measurement tasks for these crops by learning general geometric priors. Second, in outdoor environments, the main challenge lies in complex background interference and extreme lighting. In the future, introducing data augmentation strategies for complex backgrounds or using a small amount of outdoor labeled data for fine-tuning during the transfer learning phase can extend the model’s application from controlled greenhouses to open-field environments.

In summary, the organ-scale phenotyping analysis framework constructed in this study provides technical support for connecting greenhouse perception data with precision agricultural management decisions. In future work, research will focus on selecting multi-view fusion to reconstruct complete plant models to overcome occlusion issues from single-view perspectives. Diverse greenhouse crops will be selected as data samples to enhance the model’s generalization ability. Additionally, an automated preprocessing pipeline will be constructed in conjunction with lightweight networks to achieve end-to-end real-time processing from data acquisition to parameter output. However, whether in segmentation or phenotype calculation, errors will propagate. They will accumulate and amplify during batch data processing, making them inevitable. Therefore, the model and calculation methods will be further optimized in the future.

## 4 Conclusions

In this research preparation stage, we have improved the sampling strategy of the sampling layer and proposed VGDS-PointNet++. This model performed an average time consumption of 0.132 s during downsampling and realized the automatic division of three characteristic areas of the leaf, the top of the canopy, and the stem. The average segmentation accuracy was 96.80%, and the mIoU was 88.95%. An effective tomato organ segmentation method is proposed. Based on characteristics of the unclosed point cloud, the least squares curve fitting was applied to three-dimensional space by combining the principle of plane projection, the principle of slicing, and the curve fitting method. The spatial curves of the tomato leaf point cloud were extracted, and the length of the leaf was calculated with a coefficient of determination of 0.93. The stem diameter of the plant was calculated using methods such as point cloud slicing and OBB; the coefficient of determination was 0.87. There was a significant correlation between both the calculated and measured values. This method is able to obtain the leaf length more accurately.

VGDS-PointNet++ performed significant improvements in time complexity and segmentation accuracy. It laid a solid foundation for subsequent fruit segmentation and phenotypic calculation. There is room for improvement not only in the sampling layer but also in the organizational layer, feature extraction layer, and others. Future work will consider optimizing these other layers to better adapt to different types of data and enhance the model’s generalization capabilities. Alternative statistical approaches incorporating non-linear modeling or machine learning algorithms could be employed to better capture the complex dynamics of leaf growth. Advanced non-linear regression techniques or deep neural networks may enhance both model fitting precision and predictive performance.

## Data Availability

The raw data supporting the conclusions of this article will be made available by the authors, without undue reservation.
